# Influence of Sense of Competence, Empathy and Relationship Quality on Burden in Dementia Caregivers: A 15 Months Longitudinal Study

**DOI:** 10.1177/07334648221138545

**Published:** 2022-11-16

**Authors:** Robin van den Kieboom, Ruth Mark, Liselore Snaphaan, Marcel van Assen, Inge Bongers

**Affiliations:** 1 Tranzo, Tilburg School of Social and Behavioral Sciences, Tilburg University, Tilburg, The Netherlands; 2 Research Unit Evidence Based Management of Innovation, Mental Health Care Institute Eindhoven, Eindhoven, The Netherlands; 3 Department of Cognitive Neuropsychology, Tilburg School of Social and Behavioral Sciences, Tilburg University, Tilburg, The Netherlands; 4 Department of Methodology and Statistics, Tilburg School of Social and Behavioral Sciences, Tilburg University, Tilburg, The Netherlands; 5 Department of Sociology, Utrecht University, Utrecht, The Netherlands

**Keywords:** dementia, caregiving, longitudinal methods

## Abstract

**Objectives:** The aim is to explore the trajectory of caregiver burden and how this relates to caregiver and contextual factors in community-dwelling dyads. **Methods:** At baseline, 201 family caregivers were included. The multidimensional construct of family caregiver burden and the effects of sense of competence, empathy, and quality of the relationship on this burden were assessed over 15 months using semi-structured interviews and questionnaires. **Results:** We found an increase of burden linked to disruptions in the caregiver’s own usual activities (*p* = 0.002) and physical health complaints (*p* = 0.001). Caregivers with a high sense of competence experienced lower caregiver burden during the entire caregiving process (*p* < 0.001). **Discussion:** Healthcare professionals should alert family caregivers to the importance of taking care of themselves as early as possible in their new caregiver role. Caregiving is demanding and could negatively influence their own activities and physical health.

**What this paper adds**

• By assessing the multidimensionality of caregiver burden, this paper provides insights into how different aspects of burden develop over time.
• Specific aspects of caregiver burden gradually increase over a 15-month follow-up period. Caregivers report more interruptions in their own activities and experience more physical health complaints due to the demands of caregiving.
• Sense of competence is a strong and consistent predictor for caregiver burden over time.

**Applications of study findings**

• Health care professionals need to evaluate the burden experienced by the family caregiver in a timely, multidimensional way throughout the dementia process.
• Early detection of family caregivers with a low sense of competence is crucial and interventions to address this are essential to prevent high levels of caregiver burden.
• Interventions aimed at both enhancing the physical health of the family caregiver and in enabling them to maintain their own, usual activities are recommended.

## Introduction

The majority of the approximately 50 million persons with dementia worldwide (Alzheimers Disease International, 2018) is cared for at home by their family caregivers, typically their spouses or children (Etters et al., 2008). This caregiving requires effort and time and is often described as burdensome (Pearlin et al., 1990). This caregiver burden is both a complex and a multidimensional construct (Zarit et al., 1986). Burden and its associated risk factors in family caregivers caring for people with dementia have been investigated in numerous studies (Brodaty et al., 2014; Conde-Sala et al., 2014; Viñas-Diez et al., 2017). The risk factors for caregiver burden can be clustered into three main categories, namely, factors associated with the patient, the caregiver and the context (van der Lee et al., 2014).

Caregiver factors like personality, coping style, and sense of competence all influence how or even if the caregiver will adapt to the demands of caregiving (van der Lee et al., 2014). They also mediate between the impact of the person with dementia’s neuropsychiatric symptoms and burden the caregiver might experience. Sense of competence has emerged as an important caregiver factor in recent literature (van der Lee et al., 2014) and is typically defined as the capacity or potential for acting efficiently in a given context (Dolin, 2015). In the current study, it refers to the caregiver’s feeling of being competent in their caregiving role. Caregiver empathy is another factor which can influence how the caregiver appraises the caregiving situation. Empathy is defined as a complex bio-psycho-social concept made up of at least two components (Decety & Jackson, 2004). These include a cognitive component: knowing or understanding what another person is feeling and an affective or emotional component: feeling what another person feels (Jütten et al., 2017). Several studies (Lee et al., 2001; Sutter et al., 2014) found that family caregivers with higher levels of cognitive empathy experienced less stress in their role.

Context factors, including, for example, time since diagnosis, cohabitating and the number of hours of caregiving are also associated with caregiver burden (Kim et al., 2012; Quinn et al., 2009). The quality and type of the relationship between the person with dementia and caregiver is also considered to be an important factor (Quinn, et al., 2009). Family conflicts can arise because of the caregiving situation. (Neufeld & Harrison, 2003). Steadman et al. (2007) found that caregivers who were highly satisfied with the quality of the relationship, had significantly less burden and also reacted less negatively to the memory and behavior problems exhibited by the person with dementia.

To date, most studies on caregiver burden are cross-sectional in nature despite the fact that dementia is progressive and the situation, per definition, is very likely to change over time (Agüera-Ortiz et al., 2010). A recent systematic review explored the trajectory of caregiver burden (van den Kieboom et al., 2020) and found fluctuations in caregiver burden over time. These fluctuations depended on the presence of various risk factors, such as neuropsychiatric symptoms (Conde-Sala et al., 2014) and functional decline of the person with dementia (Conde-Sala et al., 2014; Viñas-Diez et al., 2017), female gender of the caregiver (Viñas-Diez et al., 2017), and the type of relationship between the dyads (Conde-Sala et al., 2014).

Previous longitudinal studies have typically focused on the person with dementia and explored which of these factors were linked to an increased risk for caregiver burden. However, aforementioned cross-sectional research showed that caregiver—and context characteristics were also strongly related to caregiver burden. To date, one longitudinal study (van der Lee et al., 2017) reported that the caregiver’s sense of competence was an important factor for caregiver burden over time. Caregivers who felt less competent, reported more burden over time. Another longitudinal study reported that caregivers with poor family functioning at initial assessment had higher ratings of strain and burden after 1 year (Heru & Ryan, 2006).

More longitudinal research is needed in order to explore how caregiver factors, like sense of competence and empathy and context characteristics like the quality of the relationship influence caregiver burden as the dementia progresses. With these insights, clinical practice may be able to intervene on these predictors to prevent elevated levels of burden in family caregivers.

This study has three aims: (a) to assess the development of the different dimensions of caregiver burden over time, (b) to explore whether the caregiver’s sense of competence, empathy and the quality of their relationship with the person with dementia affects caregiver burden, and (c) to explore whether the effect of these predictors on burden changes over a 15-month follow-up period.

## Methods

### Study Sample

This study used the existing data set of the longitudinal quasi-experimental Into D’mentia study (Jütten et al., 2017). The original study explored whether a mixed virtual reality simulator increased family caregivers’ understanding for people with dementia, their empathy, sense of competence, relationship quality with the care receiver, and/or decreased burden, depression, and anxiety. In total, 267 participants were screened for eligibility and 201 were included (see Supplementary file 1 for the inclusion/exclusion Flow Chart). All participants were caregivers of a person with dementia who was also living at home. Drop-outs were due to deterioration in the person with dementia’s status (33.3%), institutionalization of the person with dementia (30.7%), or no reason was given (36.0%). The participants who did not complete an assessment without giving reasons why, were nevertheless invited to take part in the follow-up measurements.

All participants were adult family caregivers (who spent at least 8 hours per week on caregiving) of a relative, spouse, or friend with dementia who lived at home. Participants were excluded if they had severe communication difficulties (like insufficient understanding of the Dutch language, blindness, or deafness) or severe psychological or medical disabilities. The recruitment of participants started in July 2014 and ended in January 2017.

### Measurements

The Caregiver Reaction Assessment Dutch (CRA-D) was used to assess caregiver burden (Given et al., 1992). The CRA consists of 24 items that contain four negative dimensions and one positive dimension. These include: Disrupted Schedule (DS), Financial Problems (FP), Family Support (FS), Physical Strength (PS), and Self-Esteem (SE). The total scores on all five dimensions were outcome variables in the current study.

The subscale Disrupted Schedule (DS) (5 items, α = 0.86) measures the extent to which caregiving interrupts the usual activities of the caregiver. An example item is: *“My activities are centered around care for…”* The subscale Financial Problems (FP) (3 items, α = 0.91) measures the financial strain on the caregiver as a consequence of the caregiving situation. An example item is: “*Caring for …. has put a financial strain on the family.”* The subscale Family Support (FS) (4 items, α = 0.83) assesses the extent to which the caregiver perceives a shortage of family support and the caregiver’s perception of being abandoned in their caregiving responsibilities. An example item is: *“Since caring for…, I feel my family has abandoned me.”* The subscale Physical Strength (PS) (5 items, α = 0.83) assesses the caregiver’s feeling of deterioration in physical health. An example item is: *“My health has gotten worse since I’ve been caring for…”* The subscale Caregiver’s Self-Esteem (SE) (7 items, α = 0.80) aims to measure the extent to which caregiving contributes to individual self-esteem. An example item is: *“I enjoy caring for…”* Caregivers were asked to rate the perceived impact of caregiving on a 5-point Likert scale. The range of possible scores for each subscale is as follows; Disrupted Schedule (5–25), Financial Problems (3–15), Family Support (4–20), Physical Strength (5–25), and Self-Esteem (7–35). For the first four subscales a lower score indicates a lower subjective burden. For the fifth and final subscale, Self-Esteem, a higher score indicates a lower subjective burden.

### Caregiver Predictors

The Short Sense of Competence Questionnaire (SSCQ) consists of seven items, each rated according to a 5-point Likert scale (1–5). The total score ranges from 0 to 35, with higher scores indicating more sense of competence (Vernooij-Dassen et al., 1999). In this study, the SSCQ had a Cronbach’s alpha of 0.71.

The Interpersonal Reactivity Index (IRI) was used to measure empathy (Davis, 1983). The IRI asks subjects to rate 28 items on several empathy-related statements on a 5-point Likert scale (1–5) ranging from “does not describe me well (1)” to “describes me very well (5).” The 28 items are clustered into four subscales, each with seven different items, namely, perspective taking (PT), fantasy (FS), empathic concern (EC), and personal distress (PD). The Cronbach’s alpha for the subscales ranged from 0.69 to 0.80. The subscales EC and PD are combined to represent the “affective empathy” factor and the subscales PT and FS into the “cognitive empathy” factor of the IRI (Stocks & Lishner, 2017). In this study, “affective empathy” and “cognitive empathy” were both included as predictors. In this study, the Cronbach’s alpha for the affective empathy factor was 0.75 and for the cognitive empathy factor it was 0.74

Caregiver sociodemographic variables were collected in an interview at baseline, and included: age, gender (male or female), educational level (7 levels (Verhage, 1964), type of relationship (spouse, adult child, friend or acquaintances, others), the number of days and hours providing care per week, and currently employed (yes or no).

### Context Predictor

Relationship quality between caregiver and the person with dementia was evaluated using a questionnaire based on the Affectual Solidarity Questionnaire used for the Longitudinal Study of Generations (Silverstein & Bengtson, 2019). In the current study, this is referred to as Quality of the Relationship (QoR). This questionnaire evaluates the current relationship quality with five items on a 4-point scale. Scores range from 5 to 20, with a higher score indicating a better relationship quality. The Cronbach’s alpha was 0.81 in this study.

### Person with Dementia Predictors

Clinical variables of the people with dementia were collected from the caregivers during an interview conducted during the baseline measurement. These included: the diagnosis given (Alzheimer’s disease, vascular dementia, Parkinson dementia, other or unknown), the time since diagnosis in years, and the living situation (alone, with spouse, other relatives, others or institutionalized).

### Procedure

All participants completed a semi-structured interview and a questionnaire booklet at the four time points: baseline (T1), 1 week (T2), 2.5 months (T3), and 15 months (T4). The questionnaire booklet was sent to the participants before the appointment for the interview. The interviews were administered in a standardized way by trained neuropsychologists and took place either at the participant’s home or at the university depending on the caregivers’ preference. This study was carried out in agreement with the Declaration of Helsinki. Written informed patient consent was obtained.

### Statistical Analysis

Statistical analyses were performed using SPSS Statistics 27. Descriptive statistics (means and standard deviations or frequencies) were used to summarize the sociodemographic variables of the caregiver and clinical variables of the people with dementia based on non-missing observations.

To assess the development over time of caregiver burden, mixed model analyses were separately performed for each of the five subscales of the CRA, with time as factor (four levels) as well as linear in measurement (assuming faster change early on, as later measurements are further apart). To control for multiple testing, alpha was therefore set at 0.05/5 = 0.01 for all statistical analyses. Effect sizes were calculated by subtracting the mean of the subscales of the CRA at baseline from the mean at 15 months and subsequently dividing this outcome by the standard deviation at baseline. To account for missing values (Supplementary file 2) on predictors included in the mixed model analysis, multiple imputation (Sterne et al., 2009) was used in SPSS 27. Importantly, we only applied multiple imputation to the predictors, as mixed model analysis already adequately deals with missingness in the outcome measures (e.g., Twisk, 2013). It aims to allow for the uncertainty about the missing data by creating several different plausible imputed data sets and appropriately combining results obtained from each of them. The number of imputations was set to 20 (Horton & Lipsitz, 2001) and the model for scale variables was linear regression.

Bivariate correlations were conducted in order to explore whether any of the associations between the four predictors were significant. In the second set of mixed model analyses, four main predictors (sense of competence, affective empathy, cognitive empathy, and quality of the relationship on burden to the model) were added, as well as some covariates. These covariates were selected based on statistically significant bivariate correlations (by exception α = 0.05) between the individual CRA subscales and person with dementia baseline characteristics.

In the third and last set of mixed model analyses, separate interactions terms of time with the predictors were added one by one to explore differences in the effects of the predictors on burden. If an interaction was statistically significant, simple effect analyses were performed to assess the effect of the predictors for each point in time. The fit of the three models was assessed and compared using the likelihood ratio test. For testing continuous and categorical predictors we applied *t*- and *F*-tests, respectively.

## Results

### Sample Description

The demographics of the family caregivers and clinical characteristics of the people with dementia are presented in Table 1. In this study, most of the caregivers were female, well-educated and the majority were adult children or spouses. On average, caregivers were younger compared to the people with dementia and most of the caregivers were employed. On entry to this study, the average years of caregiving was about 4 years and the hours of care per week provided was over 50 hours. The majority of the people with dementia had Alzheimer’s disease and most lived with their spouses.

**Table 1.** Demographics of informal caregivers and clinical characteristics of the people with dementia.

Total sample N = 201 Variable (n)	Mean (SD)	Frequency (%)
Caregiver		
Age in years (192)	60.83 (12.03)	
Gender (201)		
Male		21.4%
Female		78.6%
Educational level^1^ (200)		
Low		14.9%
Medium		35.8%
High		48.4%
Current employed (201)		
Yes		67.7%
No		32.3%
Years of caregiving (200)	3.86 (3.15)	
Hours of care per week (199)	56.02 (63.08)	
Relationship with the person with dementia (200)		
Spouse		41.0%
Children		45.0%
Friends or acquaintance		0.5%
Other		13.5%
People with dementia		
Age (189)	78.6 (8.29)	
Gender (198)		
Male		43.9%
Female		56.1%
Time since diagnose (193)	3.14 (2.49)	
Diagnose (200)		
Alzheimer’s disease		58.0%
Vascular dementia		17.5%
Parkinson dementia		2.0%
Other		5.0%
Unknown		11.5%
No diagnose		4.0%
Living situation (198)		
Alone		18.2%
With spouse		60.6%
With other relatives		3.0%
Others		4.5%
Institutionalized		13.6%

^1^Educational level according to Verhage [48], recoded as low (1–4), medium (5), or high (6–7).

### The Development Over Time of Caregiver Burden

The results of the mixed model analysis with time as predictor are depicted in Table 2.

**Table 2.** Results of mixed model analyses of the CRA subscales with time as discrete predictor.

	Baseline Mean (sd)	One week Mean (sd)	2.5 Months Mean (sd)	15 Months Mean (sd)	*Sig.*	Effect Size
CRA Disrupted schedule	15.09 (4.53)	14.90 (4.34)	15.48 (4.61)	15.87 (4.82)	F (3.159) = 10.05 *p* = 0.002^a^	0.17
CRA Financial problems	7.22 (2.11)	7.17 (2.29)	7.24 (2.42)	7.49 (2.52)	F (3.*168)* = 0.36 *p* = 0.549	0.13
CRA Family support	11.78 (4.22)	11.84 (4.14)	12.43 (3.90)	12.43 (4.44)	F (3.169) = 4.38 *p* = 0.038	0.15
CRA Physical strength	10.12 (3.45)	10.04 (3.44)	10.33 (3.29)	10.84 (3.69)	F (3.162) = 10.91 *p* = 0.001^a^	0.21
CRA Self-esteem	26.26 (4.04)	26.05 (4.29)	26.06 (4.28)	25.83 (4.48)	F (3.142) = 5.09 *p =* 0.026	0.10

^a^Significant at the 0.01 level (2-tailed).

A significant increase over time was found on the subscales Disrupted Schedule (*p* = 0.002) and Physical Health (*p* = 0.001) during the 15-month follow-up period, which corresponds to small effect sizes equal to 0.10 and 0.21, respectively. Caregivers experienced more interruptions in their own usual activities and also more health complaints and physical deterioration over time. No significant changes over time were found on the other subscales of the CRA (*p* > 0.01).

Effect of caregiver’s sense of competence, empathy, and the quality of their relationship with the person with dementia on caregiver burden

The correlations between the predictors (Table 3) showed significant associations between SSQ and, respectively IRI Affective (*r* = −0.127, *p* < 0.001), IRI Cognitive (*r* = 0.097, *p* = 0.014), and QoR (*r* = 0.379, *p* < 0.001). IRI Affective and IRI Cognitive were also significantly correlated to each other (*r* = 0.832, *p* < 0.001); however, QoR was not significantly associated with IRI Affective (*r* = −0.050, *p* = 0.233) or IRI Cognitive (*r* = 0.071, *p* = 0.089).

**Table 3.** Bivariate correlations of the predictor variables.

	SSCQ	IRI Affective	IRI Cognitive	QoR
SSCQ	1	-	-	-
IRI affective	*r* (642) = −0.127 *p* < 0.001^b^	1	-	-
IRI cognitive	*r* (642) = 0.097 *p* = 0.014^a^	*r* (654) = 0.832 *p* < 0.001^b^	1	-
QoR	*r* (577) = 0.379 *p* < 0.001^b^	*r* (580) = −0.050 *p* = 0.233	*r* (580) = 0.071 *p* = 0.089	1

^a^Significant at the 0.05 level (2-tailed).

^b^Significant at the 0.01 level (2-tailed).

Based on their correlations with the subscales of the CRA and the caregiver and person with dementia baseline characteristics (Table 4), the following sociodemographic variables were included in the second set of mixed model analyses; age of the caregiver, age of the person with dementia, type of relation, educational level, and employment status of the caregiver. The results of these mixed model analyses are presented in Table 5. First adding the four predictors (sense of competence, affective empathy, cognitive empathy, and quality of the relationship) in the mixed model analysis improved the explanation of caregiver burden for all subscales (*p* < 0.001, 8^th^ row of Table 4) except Financial Problems (*p* = 0.079). Subsequently adding the sociodemographic covariates resulted in a significant better model fit for the subscales Disrupted Schedule, Financial Problems, and Physical Complaints (*p* < 0.001), but not for the subscales Family Support (*p* = 0.081) and Self-Esteem (*p* = 0.919). Overall, prediction of caregiver burden was improved for all subscales after adding the in total eight predictors (*p* < 0.001, last row of Table 4). Sense of competence was a consistent predictor for all the subscales of the CRA (*p* < 0.001), except for the subscale Financial Problems (*p* = 0.038). A better quality of the relationship predicted more feelings of self-esteem *(p* < 0.001), but had no effects on the other subscales of the CRA. Affective empathy predicted one CRA subscale, namely, Physical Strength (*p* = 0.006). Cognitive empathy did not predict any of the CRA subscales.

**Table 4.** Bivariate correlations of demographic with caregiver subscales on baseline.

	CRA Disrupted Schedule	CRA Financial Problems	CRA Lack of Family Support	CRA Loss of Physical Strength	CRA Self-Esteem
Age caregiver	*r* (192) = 0.399 ***p* < 0.001**^b^	*r* (191) = 0.006 *p* = 0.934	*r* (192) = 0.23 *p* = 0.089	*r* (192) = 0.300 ***p* < 0.001**^b^	*r* (192) = 0.055 *p* = 0.447
Gender caregiver	*r* (200)= 0.103 *p* = 0.148	*r* (199) = 0.101 *p* = 0.158	*r* (200) = 0.100 *p* = 0.160	*r* (200) = 0.039 *p* = 0.582	*r* (200) = 0.066 *p* = 0.353
Type of relation	*r* (199) = 0.373 ***p* < 0.001**^b^	*r* (198) = 0.101 *p* = 0.158	*r* (199) = 0.094 *p* = 0.188	*r* (199) = 0.271 ***p* < 0.001**^b^	*r* (199) = 0.098 *p* = 0.167
Educational level	*r* (200) = 0.112 *p* = 0.113	*r* (199) = 0.223 ***p* = 0.002**^b^	*r* (200) = 0.124 *p* = 0.080	*r* (200) = 0.126 *p* = 0.076	*r* (200) = 0.087 *p* = 0.218
Current employed	*r* (200) = 0.310 *p* < 0.001^a^	*r* (199) = 0.107 *p* = 0.133	*r* (200) = 0.145 ***p* = 0.040**^a^	*r* (200) = 0.169 ***p* = 0.017**^a^	*r* (200) = 0.090 *p* = 0.205
Time since diagnose	*r* (192) = 0.002 *p* = 0.973	*r* (191) = 0.015 *p* = 0.842	*r* (192) = 0.191 *p* = 0.08	*r* (192) = 0.008 *p* = 0.917	*r* (192) = 0.006 *p* = 0.930
Type of diagnose	*r* (199) = 0.121 *p* = 0.089	*r* (198) = 0.055 *p* = 0.440	*r* (199) = 0.026 *p* = 0.712	*r* (199) = 0.030 *p* = 0.674	*r* (199) = 0.039 *p* = 0.587
Age person with dementia	*r* (189) = 0.123 *p* = 0.092	*r* (188) = 0.237 ***p* = 0.001**^b^	*r* (189) = 0.086 *p* = 0.241	*r* (189) = 0.036 *p* = 0.619	*r* (189) = 0.072 *p* = 0.322
Living situation patient	*r* (197) = 0.071 *p* = 0.320	*r* (196) = 0.109 *p* = 0.129	*r* (197) = 0.110 *p* = 0.122	*r* (197) = 0.103 *p* = 0.148	*r* (197) = 0.104 *p* = 0.145
Gender person with dementia	*r* (197) = 0.118 *p* = 0.099	*r* (196) = 0.110 *p* = 0.124	*r* (197) = 0.080 *p* = 0.263	*r* (197) = 0.072 *p* = 0.313	*r* (197) = 0.122 *p* = 0.089

^a^Significant at the 0.05 level (2-tailed).

^b^Significant at the 0.01 level (2-tailed).

**Table 5.** Results of mixed model analysis of the CRA subscales with predictors and covariates (time linear in measurement).

	CRA Disrupted Schedule b [95% CI] *t(df) p*	CRA Financial Problems b [95% CI] *t(df) p*	CRA Family Support b [95% CI] *t(df) p*	CRA Physical Strength b [95% CI] *t (df)* *p*	CRA Self-Esteem b [95% CI] *t (df) p*
Intercept	14.618 [13.958/15.280] *t(199)* = 43.597 ***p* < 0.001**^a^	7.166 [6.811/7.521] *t(199)* = 39.798 ***p* < 0.001**^a^	11.579 [10.916/12.243] *t(203)* = 34.408 ***p* < 0.001**^a^	9.786 [9.253/10.320] *t(197)* = 36.174 ***p* < 0.001**^a^	26.496 [25.901/27.091] *t(193)* = 87.824 ***p* < 0.001**^a^
Measurement	.238 [.074/.402] *t* (*149*) = 2.871 ***p* = 0.005**^a^	.034 [−.085/.165] *t* (*168*) = 0.627 *p* = 0.531	.162 [−.004/.327] *t* (*170*) = 1.930 *p* = 0.055	.206 [0.063/.349] *t* (*154*) = 2.849 ***p* = 0.005**^a^	−.135 [−.296/.026] *t* (*136*) = −1.656 *p* = 0.100
SSCQ total	−.196 [−.249/−.144] *t(391)* = −7.329 ***p* < 0.001**^a^	−.038 [−.074/-.003] *t (500)* = −2.072 *p* = 0.038	−.101 [−.153/−.050] *t(373)* = −3.809 ***p* < 0.001**^a^	−.188 [−.231/−.146] *t(434)* = −8.728 ***p* < 0.001**^a^	.180 [.128/0.231] *t(281)* = 6.908 ***p* < 0.001**^a^
QoR total	−.063 [−.153/0.026] *t* (*577*) = −1.016 *p* = 0.310	−.016 [−.074/.043] *t(586)* = −0.610 *p* = 0.542	−.078 [−.165/.009] *t(569)* = −1.760 *p* = 0.133	-.082 [-.153/−0.12] *t(591)* = −1.926 *p* = 0.055	.224 [.131/.316] *t(585)* = 4.473 ***p* < 0.001**^a^
IRI cognitive	.032 [−.011/.074] *t(562)* = 1.425 *p* = 0.154	−.002 [−.029/.025] *t(549)* = −0.090 *p* = 0.928	−.038 [−.080/.004] *t(565)* = −1.753 *p* = 0.080	.003 [−.031/.037] *t(575)* = 0.139 *p* = 0.890	.034 [−.011/.078] *t(575)* = 1.544 *p* = 0.123
IRI affective	*.*025 [−.012/.068] *t(591)* = 1.135 *p* = 0.257	−.004 [−.031/.022] *t(530)* = −0.436 *p* = 0.663	.008 [−.033/0.051] *t(576)* = 0.386 *p* = 0.700	.047 [.014/.080] *t(570)* = 2.757 ***p* = 0.006**^a^	.038 [−.007/.082] *t(579)* = 1.590 *p* = 0.122
Improvement model fit	χ^2^ *(4) =* 78.854 ***p* < 0.001**^a^	χ^2^ *(4)* = 8.341 *p* = 0.079	χ^2^ (4) = 28.893 **p < 0.001**^a^	*χ*^*2*^ *(4) =* 113.104 *p* < 0.001^a^	*χ*^*2*^ *(4)=* 91.544 ***p* < 0.001**^a^
Age caregiver at baseline	.073 [.027/.119] *t(260)* = 2.881 *p* = 0.004**	−.018 [−.043/.007] *t(225)* = −0.728 *p* = 0.467	.028 [−.020/0.075] *t(274)* = 1.119 *p* = 0.264	.040 [.006/0.074] *t(246)* = 2.134 ***p* = 0.033**^a^	.004 [−.039/.047] *t(247)* = 0.584 *p* = 0.560
Age person with dementia at baseline	−.046 [−.096/.004] *t* (*336*) = −0.493 *p* = 0.622	−.041 [−.069/−.012] *t(274)* = −3.006 ***p* = 0.003**^a^	−.037 [−.089/.015] *t(378)* = −0.376 *p* = 0.707	−.015 [−.053/.023] *t(311)* = −0.697 *p* = 0.486	.036 [−.013/.085] *t(292)* = 0.507 *p* = 0.612
Type of relation	F (3,642) = 7.575 ***p* = 0.006**^a^	F (*3,642*) = 0.681 *p* = 0.410	F (*3,642*) = 0.015 *p* = 0.901	F(3,642) = 2.449 *p* = 0.119	F(3,642) = 0.045 *p* = 0.832
Educational level	F(2,642) = 0.010 *p* = 0.972	F (*2,642*) = 8.811 ***p* = 0.003**^a^	F (*2 642*)= 0.398 *p* = 0.529	F(2,642) = 1.082 *p* = 0.300	F(2,642) = 0.748 *p* = 0.388
Employment status	F (*1,644*) = 1.815 *p* = 0.175	F (*1,644*) = 0.248 *p* = 0.619	F (1,644) = 0.161 *p* = 0.688	F(1,644) = 0.235 *p* = 0.628	F(1,644) = 0.954 *p* = 0.330>
Improvement model fit	χ^2^ *(5)* = 45.348 ***p* < 0.001**^a^	χ^2^ *(5)* =24.576 ***p* < 0.001**^a^	χ^2^ *(5)* = 9.797 *p* = 0.081	χ^2^ *(5)* = 24.819 ***p* < 0.001**^a^	χ^2^ *(5)* = 0.149 *p* = 0.919
Improvement model fit (predictors + covariates)	χ^2^ *(9)* = 135.663 ***p* < 0.001**^a^	χ^2^ *(9)* = 33.276 ***p* < 0.001**^a^	χ^2^ *(9)* = 42.885 **p < 0.001**^a^	χ^2^ *(9)* = 148.124 ***p* < 0.001**^a^	χ^2^ *(9)* = 96.571 ***p* < 0.001**^a^

^a^Significant at the 0.01 level (2-tailed).

Regarding sociodemographic variables, age of the caregiver on entry of the study was positively related to burden only on the subscale Disrupted Schedule (*p* = 0.004), whereas age of the person with dementia on entry of the study was negatively related to financial problems experienced by the caregiver (*p* = 0.003). The older the caregiver, the more the caregiving disrupted their usual activities and the younger the caregiver, the higher the impact of care giving on their financial situation. Type of relation (*p* = 0.006) was associated with more burden on the subscale Disrupted Schedule; spouses reported more burden on this domain compared with the children of the people with dementia and others. Educational level of the caregiver was a significant predictor (*p* = 0.003) on the subscale of Financial Problems; caregivers with a high level of education experienced less burden on this domain compared with caregivers with a low or medium level of education. Employment status was not related with any of the subscales of the CRA.

### The Influence of the Predictors on Burden Changes Over Time

To explore whether the effects of the predictors on the subscales of the CRA varied over time, the interaction between time of measurement and the predictors were included in the mixed model analyses (Table 6). Only the effect of the Quality of the Relationship on the subscale Self-Esteem varied over time (*p* = 0.004). Although it remained significant at each point in time (*p* < 0.001), the effect decreased during follow-up (baseline estimate QoR = 0.264 versus estimate 0.184 at 15 months).

**Table 6.** Results of mixed model analysis of the caregiver subscales with interaction of time with the individual predictors. Interactions were added separately.

	CRA Disrupted Schedule	CRA Financial Problems	CRA Family Support	CRA Physical Strength	CRA Self-Esteem
Measurement × SSCQ	F(3,186) = 2.671 *p* = 0.104	F(3,246) = 0.361 *p* = 0.548	F(3,223) = 0.107 *p* = 0.744	F(3,208) = 5.366 *p* = 0.022	F(3,166) = 0.009 *p* = 0.923
Measurement × QoR	F(3,202) = 1.197 *p* = 0.275	F(3,252) = 2.917 *p* = 0.089	F(3,221) = 3.313 *p* = 0.070	F(3,218) = 5.196 *p* = 0.024	F(3,167) = 8.447 *p* = 0.004*
Measurement × IRI Cognitive	F(3,170) = 0.590 *p* = 0.443	F(3,224) = 0.909 *p* = 0.341	F(3,205) = 2.143 *p* = 0.145	F(3,192) = 0.176 *p* = 0.675	F(3,157) = 2.163 *p* = 0.143
Measurement × IRI Affective	F(3,174) = 0.091 *p* = 0.763	F(3,210) = 0.540 *p* = 0.463	F(3,200) = 0.535 *p* = 0.465	F(3,86) = 0.006 *p* = 0.939	F(3,152) = 0.064 *p* = 0.800

* Significant at the 0.01 level (2-tailed)

## Discussion

The objective of this study was three-fold; (a) to assess the development of the different dimensions of caregiver burden over time, (b) to explore whether the caregiver’s sense of competence, empathy, and the quality of their relationship with the person with dementia affected caregiver burden, and (c) to explore whether these effects of these predictors varied over time during the caregiving process.

The results of this study showed that several dimensions of caregiver burden increased significantly over a follow-up period of 15 months. Caregivers gradually experienced more interruptions in their usual activities and more deterioration in their own physical health during the caregiving process. However, the effect sizes were small and therefore have minor clinical relevance. Although the financial strain on the caregiver and the lack of family support tended to increase over time and feelings of self-esteem decreased, these trends did not reach significance. The observed trend in this study is in line with previous research (Conde-Sala et al., 2014; Kawaharada et al., 2019), as these studies also reported a gradual increase of caregiver burden with different follow-up periods ranging from 6 to 36 months. However, a comprehensive comparison between the current study and previous research is complicated because previous studies used different instruments with a total score. Therefore, unlike the current study, they did not explore caregiver burden in a multidimensional way.

Sense of competence was a consistent predictor for four of the five aspects of caregiver burden (not for financial problems) in this study. This is in line with van der Lee et al. (2017) who also measured caregiver burden over time using a visual analog scale from the Caregiver Strain Index (Van Exel et al., 2004). Caregivers who felt more competent in their ability to care for the people with dementia, experienced less burden and more feelings of self-esteem during the caregiving process. The effect of sense of competence on burden remained stable over time and appeared to be the most important predictor of caregiver burden throughout the caregiving process. As sense of competence was a consistent predictor for caregiver burden, early detection and interventions aimed at enhancing the caregiver’s sense of competence are important. Programs such as “Partner in Balance,” which is a web-based self-management intervention, can facilitate role adaptation by supporting caregivers with finding a balance between caregiving and daily life (Boots et al., 2018) and also enhance their sense of competence.

A better quality of the relationship between the caregiver and the person with dementia predicted more feelings of self-esteem and satisfaction in the caregiving role. This is in line with previous research (Heru & Ryan, 2006; Steadman et al., 2007), in which caregivers assessing the family functioning as poor had higher ratings of strain and burden during the caregiving process. To maintain and enhance the relationship, we recommend that these dyads participate in mutual, positive activities which they both enjoy. Those activities may range from resuming previous mutual interests to starting new activities.

In this study, cognitive empathy did not predict any of the dimensions of caregiver burden. These results are in concordance with previous studies using a linear approach (Lee et al., 2001; Sutter et al., 2014). However, a previous study (Wilkinson et al., 2017) using quadratic models did find a relationship between cognitive empathy and well-being in caregivers. They found that normal levels of cognitive empathy were associated with the most depression symptoms, whereas high cognitive empathy was associated with lower depression symptoms. In the current study, affective empathy was found to have a negative effect on physical health. This relation could be mediated by anxiety and stress, as a previous study on empathy (Jütten et al., 2019) found that caregivers with more affective empathy experienced more anxiety. A review (Wilkinson et al., 2017) found that clinicians who had high levels of empathy experienced more fatigue compared with those with less affective empathy. Caregivers with more affective empathy are susceptible to more anxiety and stress and this has, in turn, been linked with more physical complaints (Turner et al., 2020)

Our explorative analyses concerning whether the effects of the predictors on caregiver burden varied over time resulted in the finding that the quality of the relationship on feelings of self-esteem became less pronounced during follow-up. A previous study (Blieszner & Shifflet, 1990) found that caregivers started to redefine their relationship with the person with dementia 6 months after diagnosis. The transition from a personal to a caring relationship may explain why the effect of the quality of the relationship in the current study became less pronounced over time on feelings of self-esteem.

The age of the caregivers affected which aspects of caregiver burden were scored higher on the multidimensional CRA. Older (compared to younger) caregivers reported not only more interruptions in their usual activities due to the caregiving but also more physical complaints. Aging itself leads to a gradual decrease in physical and mental capacity and a growing risk of disease, explaining the differences in the physical complaints. Younger caregivers, on the other hand, reported more financial problems as a result of caregiving. A possible explanation for this finding could be that spouses or children of the younger people with dementia may work themselves and the demands of caregiving prevent them from working for as many hours as they used to. Spouses reported more interruptions in their usual activities compared to adult children (or “others”) of the people with dementia but did not differ on the other domains of burden.

### Strengths and Limitations

Most studies on caregiver burden are cross-sectional in nature and this longitudinal study provides insights into the multidimensional construct of caregiver burden and the associated risk factors over a 15-month period. Longitudinal research of caregiver burden in family caregivers is still limited and extremely necessary as the current study contests. By assessing whether the effects of the associated factors change over time, an effort is made to get insight into when to intervene in the caregiving process. A limitation of the current study is the period of follow-up. The average life-expectancy after diagnosis is 3–10 years (Zanetti et al., 2009). In this study, caregivers of people with dementia entered the study on average between 3–4 years after the dementia was diagnosed. This sample of participants represents the early and midway phase of the dementia and caregiving process. By using a follow-up of 15 months, only a small fraction of the caregiving process is studied and future studies should focus on longer follow-up periods up to and including the transition of the person with dementia into a nursing home.

Inherent to longitudinal research with this population, over 50% of the initial participants dropped out by the final measurement at 15 months. The drop-out percentage is in line with previous studies (Conde-Sala et al., 2014; van der Lee et al., 2017). Furthermore, most previous studies of caregiver burden use a single total score making it impossible to explore which aspects are most/least important to caregivers. A strength of the current study is that caregiver burden was explored as a multidimensional construct. A more detailed exploration of caregiver burden was therefore possible.

Although time since diagnosis was assessed in this study, the transition to a caregiver role begins before diagnosis and the “caregiver-to-be” may already need to support the person with dementia in their daily functioning. Longitudinal research is recommended in order to explore caregiver burden and the course throughout an extended period of time starting before the dementia diagnosis.

## Conclusion

This longitudinal, multidimensional study showed that family caregivers experienced more interruptions in their own activities and reported more physical health complaints over a 15-month follow-up period due to the demands of caregiving. These findings suggest that it could be beneficial to focus on how the family caregiver appraises the caregiving situation in the early stages after (or if possible just before) the dementia diagnosis. Family caregivers should be made aware by healthcare professionals that they need to take good care of themselves right from the start (besides caring for their loved-ones) as caregiving is demanding and could negatively influence their own activities and physical health. This increased awareness could also make family caregivers more willing to share caregiving tasks and/or request help from the early stages after diagnosis and as the dementia progresses. This, in turn, could help to prevent or postpone caregiver burden which would clearly be beneficial for all concerned.

## Supplemental Material

Supplemental Material - Influence of Sense of Competence, Empathy and Relationship Quality on Burden in Dementia Caregivers: A 15 Months Longitudinal Study Click here for additional data file. Supplemental Material for Influence of Sense of Competence, Empathy and Relationship Quality on Burden in Dementia Caregivers: A 15 Months Longitudinal Study by Van den Kieboom, Robin, Mark, Ruth, Snaphaan, Liselore, van Assen, Marcel and Bongers, Inge in Journal of Applied Gerontology

Supplemental Material - Influence of sense of competence, empathy, and relationship quality on burden in dementia caregivers: A 15 months longitudinal study Click here for additional data file. Supplemental Material for Influence of sense of competence, empathy, and relationship quality on burden in dementia caregivers: A 15 months longitudinal study by Van den Kieboom, Robin, Mark, Ruth, Snaphaan, Liselore, van Assen, Marcel and Bongers, Inge in Journal of Applied Gerontology
