# Supplementary figures and images for: Influence of Sense of Competence, Empathy and Relationship Quality on Burden in Dementia Caregivers: A 15 Months Longitudinal Study
Source: J Appl Gerontol. 2022 Nov 16;42(3):464–73. doi: 10.1177/07334648221138545 (PMC9940123; doi:10.1177/07334648221138545)

**Supplementary file 1**  
**Flowchart of the participants.**

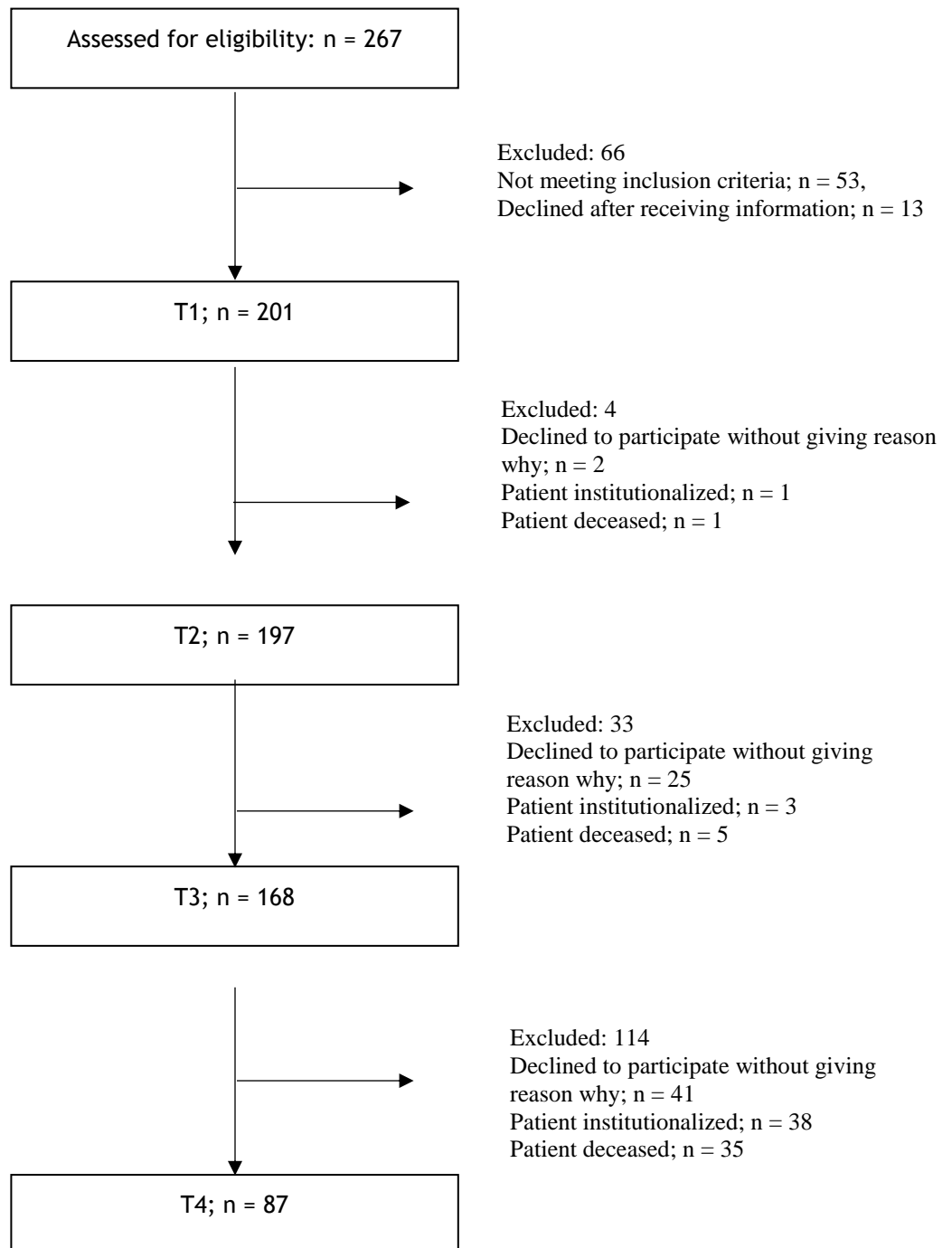

Supplement: Supplemental Material - Influence of Sense of Competence, Empathy and Relationship Quality on Burden in Dementia Caregivers: A 15 Months Longitudinal Study [file sj-pdf-1-jag-10.1177_07334648221138545.pdf]
