# Supplementary material for: Influence of Sense of Competence, Empathy and Relationship Quality on Burden in Dementia Caregivers: A 15 Months Longitudinal Study
Source: J Appl Gerontol. 2022 Nov 16;42(3):464–73. doi: 10.1177/07334648221138545 (PMC9940123; doi:10.1177/07334648221138545)
Supplement: Supplemental Material - Influence of sense of competence, empathy, and relationship quality on burden in dementia caregivers: A 15 months longitudinal study [file sj-pdf-2-jag-10.1177_07334648221138545.pdf]

Supplementary file 2
Missing data for multiple imputation

Variable	Age caregiver	Relation	Time since diagnose	Age PWD	Living situation PWD	IRI Affective	IRI Cognitive	SSCQ	QoR
Missing (n)	28	3	27	34	10	1	1	5	66
